# Palmitic Acid and Ergosta-7,22-dien-3-ol Contribute to the Apoptotic Effect and Cell Cycle Arrest of an Extract from *Marthasterias glacialis* L. in Neuroblastoma Cells

**DOI:** 10.3390/md12010054

**Published:** 2013-12-24

**Authors:** David M. Pereira, Georgina Correia-da-Silva, Patrícia Valentão, Natércia Teixeira, Paula B. Andrade

**Affiliations:** 1 REQUIMTE/Laboratory of Pharmacognosy, Department of Chemistry, Faculty of Pharmacy, University of Porto, Rua de Jorge Viterbo Ferreira, nº 228, Porto 4050-313, Portugal; E-Mails: david.ffup@gmail.com (D.M.P.); valentao@ff.up.pt (P.V.); 2 Laboratory of Biochemistry, Department of Biological Sciences, Faculty of Pharmacy, University of Porto, Rua de Jorge Viterbo Ferreira, nº 228, Porto 4050-313, Portugal; E-Mail: george@ff.up.pt; 3 IBMC—Instituto for Molecular and Cell Biology, University of Porto, Porto 4150-180, Portugal

**Keywords:** *Marthasterias glacialis* L., palmitic acid, ER-stress, CHOP, apoptosis

## Abstract

We describe the effect of a chemically characterized lipophilic extract obtained from *Marthasterias glacialis* L. against human breast cancer (MCF-7) and human neuroblastoma (SH-SY5Y) cell lines. Evaluation of DNA synthesis revealed that both cell lines were markedly affected in a concentration-dependent way, the SH-SY5Y cell line being more susceptible. Cell cycle arrest was observed, an effect induced by the sterol, ergosta-7,22-dien-3-ol, present in the extract. Morphological evaluation of treated cells showed the advent of lipid droplets and chromatin condensation compatible with apoptosis, which was confirmed by the evaluation of caspase-3 and -9 activities. Palmitic acid was the main compound responsible for this apoptotic effect by a ceramide-independent mechanism that involved endoplasmic reticulum (ER)-stress with upregulation of CCAAT/-enhancer-binding protein homologous protein (CHOP).

## 1. Introduction

Nature is an indisputable source of drugs for the human pharmacotherapeutical arsenal [1,2,3]. In recent years, marine-derived drugs have received great attention, with a steady increase in the number of molecules in clinical trials [4,5,6,7].

The chemical composition of *Marthasterias glacialis* L. has been described before, and amino acids, fatty acids, carotenoids and sterols have been identified [8,9,10]. In addition, the effect of a purified extract upon several human cancer and non-cancer cells was reported [8,11], though the mechanism responsible for the anticancer activity has not been investigated.

Nowadays, there has been increasing awareness regarding the role of the endoplasmic reticulum (ER) in the homeostasis of the cell. When ER homeostasis is disturbed, the unfolded protein response (UPR) can be activated, and the associated ER stress is known to be the basis of several cellular aggressions, namely apoptosis. In order to monitor ER status, three stress sensor proteins are known: double-stranded RNA-dependent protein kinase PKR-like ER kinase (PERK), inositol-requiring 1α (IRE1α) and activating transcription factor 6 (ATF6). In the particular case of PERK, its active form phosphorylates eIF2, which inhibits protein translation. In this branch of UPR, the DNA-damage-inducible gene 153 (GADD153), also known as C/EBP homologous protein (CHOP, a member of the C/EBP transcription factor family that heterodimerizes with other C/EBPs), is upregulated, and for this reason, it is a widely used marker of ER-stress [12,13,14]. Increased levels of CHOP have been associated with pro-apoptotic effects in several cancer cell lines, an effect attributed to CHOP-mediated repression of BCL2 gene family.

In this work, we evaluated the activity of a purified lipophilic extract from *M. glacialis* on the cancer cell lines, MCF-7 (estrogen receptor positive human breast cancer cells) and SH-SY5Y (human neuroblastoma cells), and investigated the mechanism involved in cell death and cell cycle arrest. The contribution of the main compounds present in the extract (20 µM palmitic acid, 30 µM *cis* 11-eicosenoic acid, 10 µM *cis* 11,14-eicosadienoic acid and 25 µM ergosta-7,22-dien-3-ol) is discussed.

## 2. Results and Discussion

### 2.1. Screening of *M. glacialis* Lipophilic Extract Effect on Cell Morphology and DNA Synthesis

**Figure 1:**
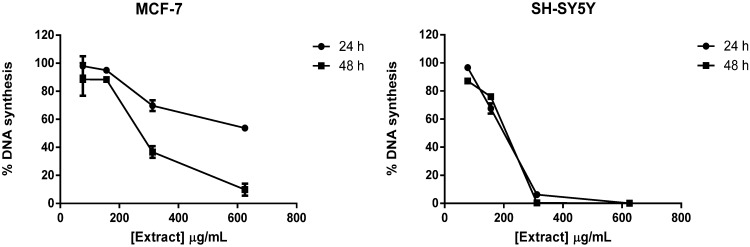
Rate of DNA synthesis in MCF-7 and SH-SY5Y cells treated with the extract (78–625 µg/mL for 24 or 48 h) by the ^3^H-thymidine incorporation assay. The results correspond to the mean ± standard deviation of three independent experiments performed in triplicate.

We evaluated the effect of a broad range of concentrations (78–625 µg/mL) of *M. glacialis* lipophilic extract in DNA synthesis. As shown in Figure 1, the extract caused a concentration-dependent reduction of DNA synthesis in both cell lines. However, in neuroblastoma cells, the effect was stronger and time-independent, while in MCF-7, it was time-dependent. These results are in line with previous reports that point to a similar behavior with regards to cell viability [11]. The lowest concentrations that elicited a biological effect were selected for morphological studies, 156 and 312 µg/mL for the SH-SY5Y and MCF-7 cell lines, respectively.

**Figure 2:**
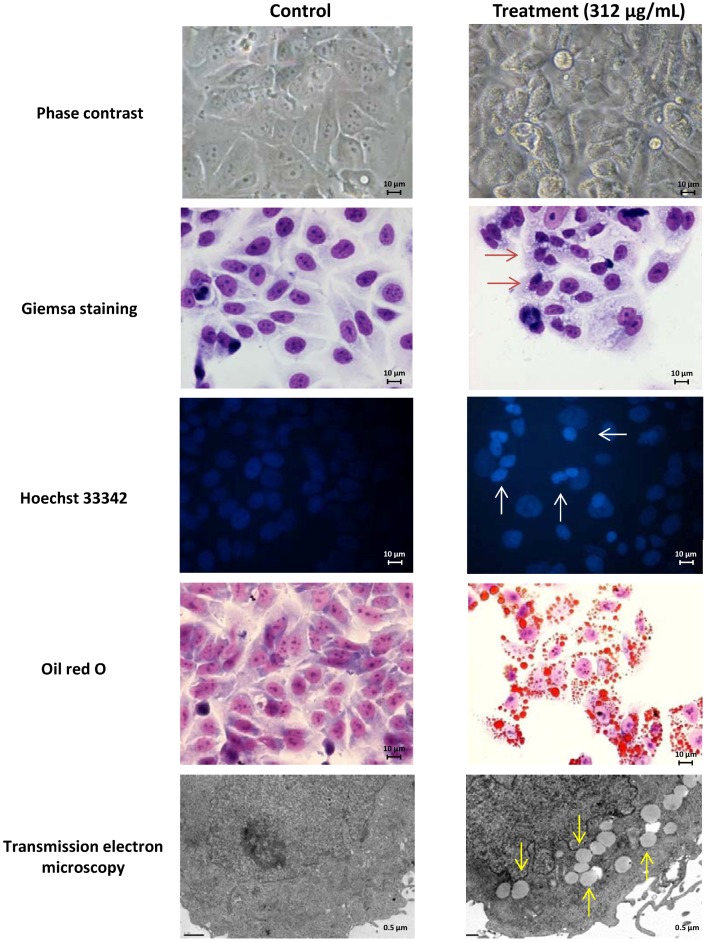
Morphological assessment of MCF-7 cells (control *vs.* treatment, 48 h of incubation). Giemsa and Hoechst 33342 stainings show chromatin condensation (red and white arrows, respectively) following incubation with the extract. Cytoplasmic vesicles, visible in Giemsa staining, proved to harbor lipophilic compounds, as shown with Oil Red O staining and transmission electron microscopy (yellow arrows).

**Figure 3:**
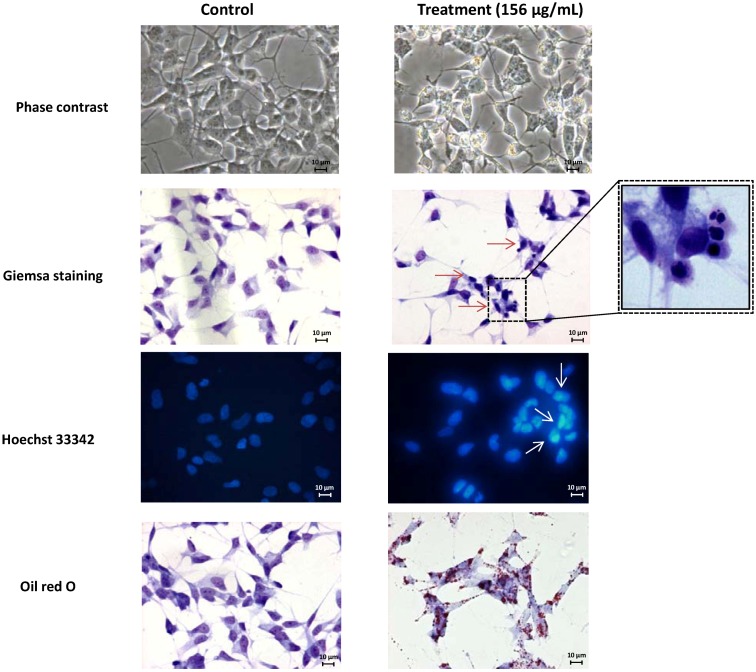
Morphological assessment of SH-SY5Y cells (control *vs.* treatment, 24 h of incubation). Giemsa and Hoechst 33342 stainings show chromatin condensation and fragmentation (red and white arrows). The advent of lipophilic cytosolic vesicles is demonstrated by the Oil Red O staining.

Several techniques were employed for the study of the effect of *M. glacialis* extract on cytoplasmic and nuclear morphology. After incubation with the extract for 48 and 24 h, both Giemsa and Hoechst 33342 stainings showed chromatin condensation in MCF-7 and SH-SY5Y, respectively, the latter presenting structures compatible with apoptotic bodies (Figure 2 and Figure 3). In both cell lines, exposure to the extract resulted in cytoplasmic vesicles. Given the lipophilic nature of the extract, we hypothesized that these vesicles could be the result of the accumulation of lipid compounds in the cell. For this reason, Oil Red O staining was performed. As shown in Figure 2 and Figure 3, Oil Red O successfully stained the cytoplasmic vesicles, which is compatible with lipid droplets. This result was further confirmed by transmission electron microscopy, which showed that these structures displayed the homogeneous grey opacity commonly found in lipid-containing organelles (Figure 2). By evaluating the effect of individual compounds on lipid droplets accumulation, we found that *cis* 11,14-eicosadienoic acid was able to elicit a similar effect regarding lipid vesicles in cytoplasm (data not shown), being likely responsible for the same effect presented by the extract. However, we cannot rule out the possibility that the accumulation of lipids in cytoplasm is related to the impact of these compounds in lipid metabolism. As the SH-SY5Y cell line showed higher susceptibility and morphological changes, it was selected for subsequent studies.

### 2.2. Ergosta-7,22-dien-3-ol Is Responsible for Cell Cycle Arrest

**Figure 4:**
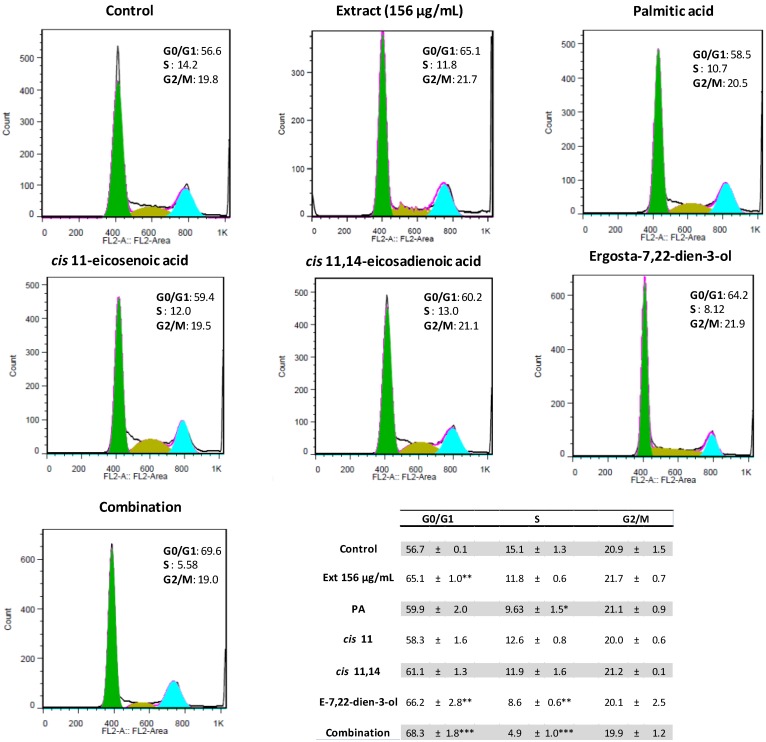
Representative histograms of the effect of *M. glacialis* lipophilic extract and pure compounds on SH-SY5Y cell cycle. The extract caused a cell cycle arrest in the G0/G1 phase, an effect attributed to ergosta-7,22-dien-3-ol. Other major compounds found in the extract had no appreciable effect. Results are expressed as the mean ± standard deviation of three experiments. * *p* < 0.05; ** *p* < 0.01; *** *p* < 0.001 (*vs.* control). PA: 20 µM palmitic acid; *cis* 11: 35 µM *cis* 11-eicosenoic acid; *cis* 11,14: 10 µM *cis* 11,14-eicosadienoic acid; E-7,22: 25 µM ergosta-7,22-dien-3-ol; combination: Palmitic acid + *cis* 11-eicosenoic acid + *cis* 11,14-eicosadienoic + ergosta-7,22-dien-3-ol.

In light of the results from the thymidine incorporation assay, we evaluated the impact of the *M. glacialis* lipophilic extract and of the pure compounds that were present in higher amounts in the extract on the neuroblastoma cells’ cell cycle. At a concentration of 156 µg/mL, the purified extract caused a G0/G1 arrest, which resulted in an increase of 10% of the number of cells in this phase (Figure 4).

In previous work, we have established the identity of major compounds and their concentrations in the purified extract from *M. glacialis* [11]. At the working concentration of 156 µg/mL, the major candidates to exert a biological effect were palmitic acid (20 µM), *cis* 11-eicosenoic acid (30 µM), *cis* 11,14-eicosadienoic acid (10 µM) and ergosta-7,22-dien-3-ol (25 µM). In order to evaluate the compounds responsible for the cell cycle arrest capacity of the extract, these compounds were evaluated individually and in combination after 24 h of incubation (Figure 4). None of the fatty acids tested showed significant effect in these values, while the sterol, ergosta-7,22-dien-3-ol, was able to cause an effect similar to that of the extract. When all compounds were tested in combination, similar values were found. Our data show that cell cycle arrest caused by the extract can be mostly attributed to the sterol, ergosta-7,22-dien-3-ol.

### 2.3. Palmitic Acid Is Responsible for the Extract-Induced Ceramide-Independent Apoptosis via the Intrinsic Pathway

**Figure 5:**
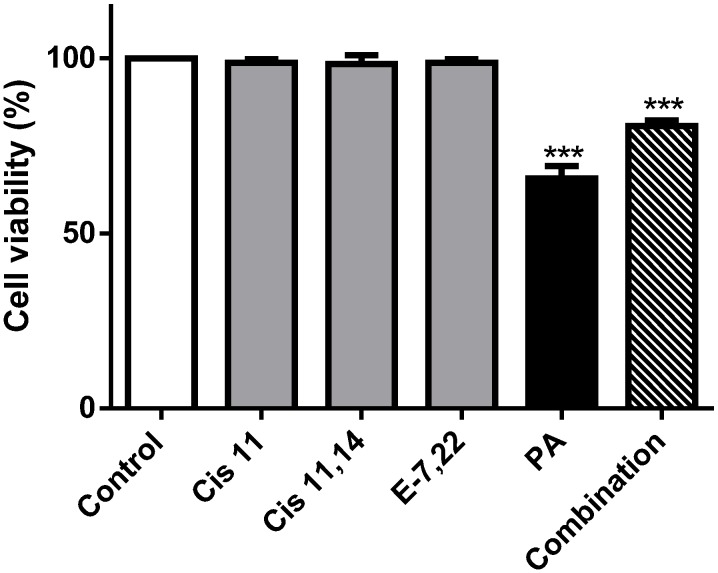
Cell viability was evaluated following incubation with the compounds present in the extract for 24 h. PA: Palmitic acid (20 µM); Cis 11: *cis* 11-eicosenoic acid (30 µM); Cis 11,14: *cis* 11,14-eicosadienoic acid (10 µM); E-7,22: Ergosta-7,22-dien-3-ol (25 µM); Combination: All compounds. Results are expressed as the mean ± standard deviation of three experiments. *** *p* < 0.001 (*vs.* control).

In previous work, we have described the impact of the purified lipophilic extract from *M. glacialis* in the cell density, viability and membrane integrity of SH-SY5Y cells [11]. At the concentration of 156 µg/mL, the extract elicited about 30% of a reduction in cell viability. Palmitic acid was the likely candidate for this activity; however, the mechanism of action was not investigated. In the work herein, we confirmed these results and showed that, apart from palmitic acid, the other compounds had no impact on cell viability. When all compounds were tested in combination, an inhibitory effect was found, albeit slightly lower than palmitic acid alone (Figure 5).

**Figure 6:**
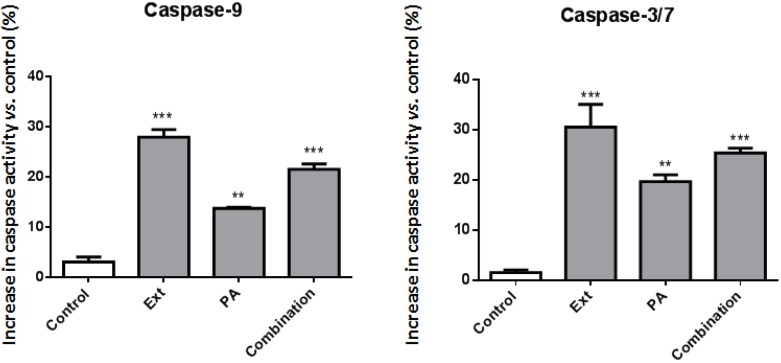
Caspase-3/-7 and -9 activity. Ext: 156 µg/mL extract; PA: Palmitic acid (20 µM); Combination: PA (20 µM) + *cis* 11-eicosenoic acid (30 µM) + *cis* 11,14-eicosadienoic acid (10 µM) + ergosta-7,22-dien-3-ol (25 µM). The results are expressed as the mean ± standard deviation of three experiments. ** *p* < 0.01; *** *p* < 0.001 (*vs.* control).

The above-mentioned morphological findings, which included apoptotic bodies in the case of SH-SY5Y cells, led us to confirm that apoptosis was taking place by evaluating the activity of caspase-3/-7, and -9. After 24 h of incubation, the extract caused an increase of about 30% in both caspase-3/-7 and -9 activity (Figure 6). While *cis* 11-eicosenoic and *cis* 11,14-eicosadienoic acids and ergosta-7,22-dien-3-ol had no impact on caspases activity (data not shown), palmitic acid elicited an increase in caspase-9 and -3/-7 activity of approximately 12 and 20%, respectively. However, when all compounds were tested in combination, higher values, closer to those displayed by the extract (18% and 25%, respectively), were found. Taken together, these results suggest that, although palmitic acid is the main compound responsible for the pro-apoptotic activity of the extract, its effect is enhanced by the presence of the unsaturated fatty acids and sterol. The pro-apoptotic effect of palmitic acid has been described in several cell lines, the concentrations used being highly dependent on cell type and as variable as 100 µM [15] and 1 mM [16]. Although our study does, indeed, use lower palmitate concentrations, it should be taken into account that the values of caspase activation found were 12% and 20%, for caspase-9 and -3, respectively.

**Figure 7:**
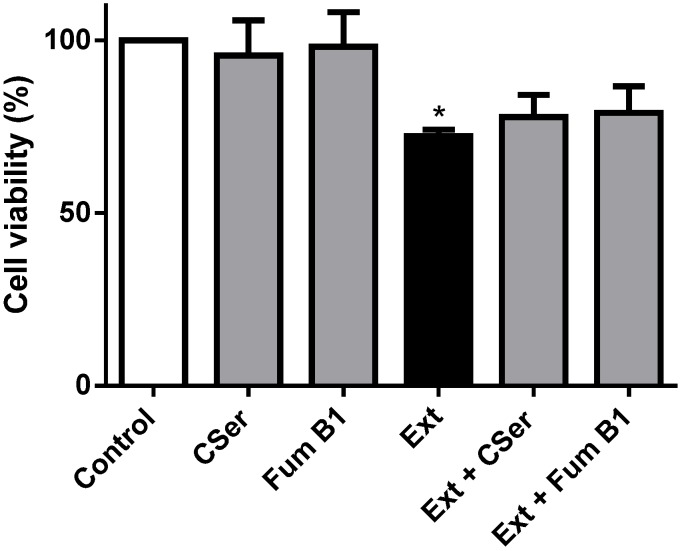
Cell viability of SH-SY5Y cells treated with the extract (Ext, 156 µg/mL) for 24 h. The ability of l-cycloserine (CSer, 500 µM) and fumonisin B1 (FumB1, 50 µM) to prevent the extract-induced loss of viability was evaluated. Both inhibitors were not cytotoxic. Results are expressed as the mean ± standard deviation of three experiments. * *p* < 0.05 (*vs.* control).

Palmitic acid plays several physiological roles in cells, from energy production to structural support in membranes. In addition, it can be the biosynthetic precursor of ceramide. Ceramide is an intra-cellular lipid signaling molecule that is involved in several cellular processes, including differentiation, growth arrest and apoptosis [17,18]. This process has been widely studied in neuronal cells [19,20]. Ceramide can be synthesized from palmitic acid via the *de novo* pathway, by the action of serine-palmitoyl transferase (SPT), and also from sphingomyelin, by the enzyme, sphingomyelinase [21]. The importance of ceramide to the pro-apoptotic effects of palmitic acid is still in discussion, and two distinct mechanisms are known: ceramide-mediated/caspase-3-independent cell death and ceramide-independent/caspase-3-dependent apoptosis [20]. We hypothesized that the high amount of palmitate in the purified extract could induce an increase of intra-cellular levels of ceramide, thus explaining the pro-apoptotic effect of the extract. In order to test this hypothesis, we evaluated the effect of two inhibitors of the key enzymes involved in the *de novo* biosynthesis of ceramide: L-cycloserine and fumonisin B1. As shown in Figure 7, the co-incubation of the extract with the two inhibitors had no significant impact on viability, and hence, we can conclude that the effects of palmitic acid are ceramide-independent. Taken together, these results show that the mechanism of cell death taking place is ceramide-independent and caspase-dependent.

### 2.4. The Extract and Palmitic Acid Cause ER-Stress Involving CHOP

**Figure 8:**
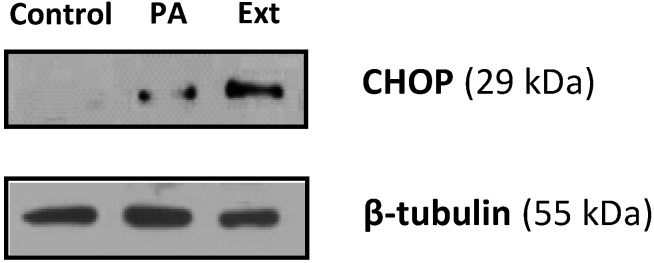
Effect of the extract (Ext, 156 µg/mL) and palmitic acid (PA, 20 µM) on the expression of CHOP by Western blot.

There has been increasing awareness regarding the role of the ER in the homeostasis of the cell. In particular, ER stress is known to be the basis of several cellular aggressions, namely apoptosis. In order to monitor ER status, several markers can be used. Nowadays, CHOP is widely used, due to its downstream involvement in several pathways of ER stress, namely PERK-eIF2a-ATF4 and ATF6 [12,13,14]. As can be found in Figure 8, incubation with the extract resulted in a marked increase of CHOP levels. Palmitic acid has been associated with ER-stress and apoptosis via ER-stress in pancreatic β-cells [16], liver cells [22] and neuronal cells [23]. Given the presence of this compound in the extract, it was also tested, and as seen in Figure 8, the CHOP protein levels were also increased in palmitic acid-treated cells. However, we cannot rule out the possibility that other compounds may contribute to the activity displayed or that other signaling pathways may also be involved in the induction of apoptosis.

**Figure 9:**
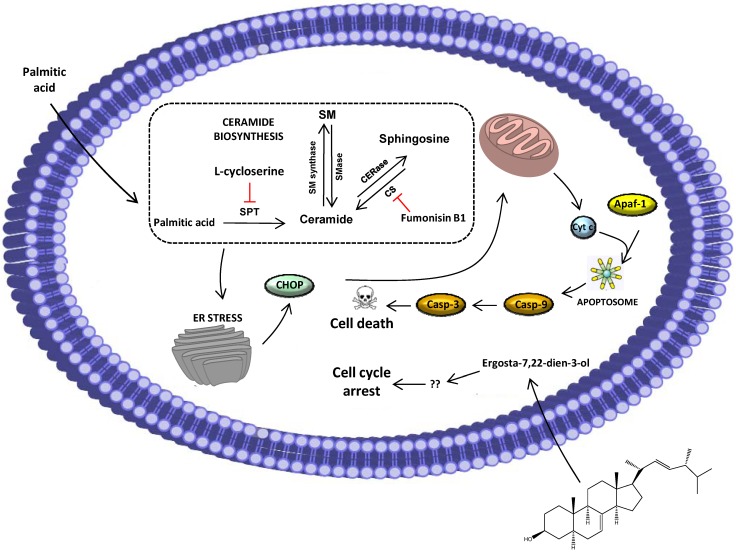
Proposed mechanism for the effect of the purified extract from *M. glacialis*. The anti-proliferative effect is caused by ergosta-7,22-dien-3-ol, which triggers cell cycle arrest. Palmitic acid is not involved in ceramide biosynthesis; instead, it causes ER-stress, as depicted by the increase in CHOP expression levels. This protein then triggers apoptosis by interacting with mitochondrial proteins. SPT: Serine palmitoyl transferase; CERase: Ceramidase; SM: Sphingomyelin; SM synthase: Sphingomyelin synthase; CS: Ceramide synthase; SMase: Sphingomyelinase; ER: Endoplasmic reticulum.

Nowadays, the link between ER stress and apoptosis has been established; however, the precise mechanisms underlying this is not completely understood. Several studies suggest that CHOP-triggered apoptosis is a result of the repression of the BCL2 gene family, thus favoring pro-apoptotic proteins, which ultimately leads to the activation of caspase-9 and, subsequently, caspase-3. This result is in line with the caspase activation reported above, and for this reason, we suggest that extract/palmitic acid-induced apoptosis is a consequence of ER-stress via the CHOP pathway (Figure 9). Although, in this work, we studied the intrinsic pathway of apoptosis, nowadays it is also known that, in some situations, CHOP can result in the activation of the extrinsic pathway as a consequence of DR5 upregulation caused by the modulation of CHOP on the promoter of this superfamily. Another player, caspase-12, has been implicated before in mice, however the fact that most humans do not possess an active caspase-12 gene [24,25] hinders our current understanding of its importance in ER-mediated apoptosis.

## 3. Experimental Section

### 3.1. Reagents and Standards

Dulbecco’s Modified Eagle Medium (DMEM), trypsin, fetal bovine serum (FBS), phosphate buffer saline (PBS), fungizone and penicillin were from GIBCO (Invitrogen, Warrington, UK). Oil Red O, glutaraldehyde, *p*-formaldehyde, DNase-free RNase A, palmitic acid (≥99%), *cis* 11-eicosenoic acid (≥99%), *cis* 11,14-eicosadienoic acid (≥98%), Triton X-100 and propidium iodide were from Sigma-Aldrich (St. Louis, MO, USA). Thymidine was from PerkinElmer (Waltham, MA, USA). Ergosta-7,22-dien-3-ol (≥98%) was from BioBioPha Co., Ltd (Yunnan, China). l-cycloserine, fumonisin B1, CHOP and β-tubulin primary antibodies, as well as anti-rabbit secondary antibody were from Santa Cruz (Heidelberg, Germany).

### 3.2. Extract Preparation

*M. glacialis* individuals were collected in Cabo Carvoeiro, Portugal, in September, 2009. Samples were frozen, transported to the laboratory, lyophilized (Labconco 4.5 Freezone apparatus, Kansas, MO, USA) and powdered using an electric blender.

The lyophilized powder (15 g) was extracted with acetone:methanol (7:3), and the extract was added to a separating funnel with 20 mL of an ether:hexane mixture (1:1). An equivalent volume of 5% NaCl was then added. The mixture was separated into two phases, and the aqueous hypophase was collected and re-extracted with the ether:hexane mixture. The organic epiphases were then collected, washed with water in order to remove traces of acetone and evaporated until dryness in a rotary evaporator. All procedures were conducted at room temperature, and the final residue was kept at −80 °C in an inert atmosphere (nitrogen).

### 3.3. Compounds Preparation

Stock solutions of all compounds were prepared in ethanol. Working concentrations were prepared by diluting stock solutions with culture medium. Controls consisting of ethanol in culture medium (0.1%–1%) were used in all cases.

### 3.4. Cell Culture

SH-SY5Y and MCF-7 cells were maintained in DMEM and MEM, respectively, with 10% FBS and 1% penicillin/streptomycin. In the case of SH-SY5Y, the cell media was also supplemented with 1% non-essential amino acids. All cells were grown in an incubator at 37 °C and 5% CO_2_.

### 3.5. Morphological Studies

For Giemsa staining cells were seeded at a density of 2 × 10^4^ cells/well in 24-well plates. After incubation with different concentrations of the extract, cells were washed twice with PBS and fixed with cold methanol, at 4 °C, for 30 min. Diluted Giemsa dye (1:10) was then added and kept for 20 min, after which cells were repeatedly washed and then mounted in DPX mountant (Sigma-Aldrich, St. Louis, MO, USA).

Changes in nuclear morphology were studied by employing Hoechst 33342 staining. Cells were fixed as described for Giemsa staining, but 4% *p*-formaldehyde was used as the fixing agent. Cells were exposed to 0.5 mg/mL Hoechst 33342 for 20 min at room temperature and mounted in Vectashield mounting medium. Preparations were examined under a fluorescence microscope (Eclipse E400, Nikon, Japan), equipped with an excitation filter with maximum transmission at 360/400 nm, and processed by Nikon ACT-2U image software.

In order to study lipid bodies, methanol-fixed cells were rinsed with propylene glycol and exposed to Oil Red O (0.7% in propylene glycol) for 7 min, with agitation. Oil red O solution was removed, and 85% propylene glycol was added and maintained for 3 min, after which, cells were washed with distilled water and mounted with aqueous mounting media.

For electron microscopy studies, cells were harvested by trypsinization after 24 and 48 h of incubation with the extract, washed with phosphate buffer fixed with 1.25% glutaraldehyde/4% *p*-formaldehyde and preserved at 4 °C for further processing. The cells were post-fixed in 1% osmium tetroxide in the same buffer, dehydrated in graded alcohols and embedded in Epon 812. Ultra-thin sections obtained with a Reichert Supra Nova ultra-microtome were collected on copper grids, stained with uranyl acetate/lead citrate and examined in a Zeiss 902A transmission electron microscope.

### 3.6. ^3^*H*-Thymidine Incorporation Assay

Cells were seeded in 96-well plates (2 × 10^4^/well) and incubated with different concentrations of extract. Incubations were maintained for 24–48 h, and for each exposure time, ^3^H-thymidine (0.5 μCi) was added to each well and incubated at 37 °C in 5% CO_2_ for the last 8 h. After two cycles of freezing/thawing, cells were harvested using a cell harvester (Skatron Instruments, Brønnøysund, Norway), and a 1 mL scintillation cocktail was added. Incorporated ^3^H-thymidine was determined in a scintillation counter (LS 6500, Beckman Instruments, Fullerton, CA, USA). Assays were carried out in triplicate, and the results are representative of three independent experiments.

### 3.7. Cell Cycle Analysis

Cell cycle analysis was performed by flow cytometry. Cells were seeded in 6-well plates (7 × 10^5^/well) and cultured with or without extract at different concentrations or with the compounds. After 24 h of treatment, cells were harvested using 0.25% trypsin in ethylenediamine tetraacetic acid (EDTA), washed twice with PBS and fixed in 70% cold ethanol. Fixed cells were finally re-suspended in 0.5 mL DNA staining solution (5 μg/mL iodide propidium [PI], 0.1% Triton X-100 and 200 μg/mL DNase-free RNase A in PBS) and kept 30 min at room temperature, in the dark.

Flow cytometric analysis of DNA content was based on the acquisition of 20,000 events in a Becton Dickinson FACSCalibur (San Jose, CA, USA) equipped with CELLQuest Pro software. Debris, cell doublets, sub-G1 population and aggregates were gated out using a two-parameter plot of FL-2-Area to FL-2-Width of PI fluorescence. Detectors for forward and side light scatter (FSC and SSC, respectively) and the three fluorescence channels (FL-1, FL-2 and FL-3) were set on a linear scale. Cell cycle histograms were analyzed using FlowJo Software (Tree Star, Inc., Ashland, OR, USA). Assays were carried out in triplicate, and the results are representative of three independent experiments.

### 3.8. Cell Viability

Cells were cultured in 96-well plates (2 × 10^4^ cells/well) and allowed to attach for 24 h. Cells were pre-incubated with the extract/compounds for 2 h, after which, lipopolysaccharide (LPS) was added and further incubated for 24 h. After incubation, 3-(4,5-dimethylthiazol-2-yl)-2,5-diphenyltetrazolium bromide (MTT) (0.5 mg/mL, final concentration) was added to each well and incubated for 2 h at 37 °C. The formazan was dissolved by the addition of a dimethyl sulfoxide (DMSO): isopropanol mixture (3:1) and quantified spectrophotometrically at 560 nm. The results of cell viability correspond to the mean of three independent experiments performed in triplicate and are expressed as the percentage of the untreated control cells.

### 3.9. Caspase-3/-7 and -9 Activity Assay

For the evaluation of caspase-3/-7 and -9 activity, the luminescent assay kits, Caspase-GloH 9, and Caspase-GloH 3/7 (Promega Corporation, Fitchburg, WI, USA), were used. Cells were seeded in white 96-well plates in the conditions reported above for MTT. As positive control, cells were incubated with staurosporine (STS) (1 µM) for 12 h. Luminescence was measured in a 96-well Microplate Luminometer (BioTek Instruments) and presented as relative light units (RLU). Assays were carried out in triplicate, and the results are representative of three independent experiments.

### 3.10. Western Blot

Cells were seeded in 6-well plates with a density of 3.5 × 10^5^ cells/well. Cells were treated for 24 h with the extract or palmitic acid. After this period, cells were washed with PBS, scraped and incubated with a lysis solution (20 mM Tris-HCl, 150 mM NaCl, 5 mM EDTA, 1% Triton) and a protease inhibitors cocktail (1 mM 4-(2-aminoethyl)benzenesulfonyl fluoride (AEBSF), 15 µM pepstatin A, 14 µM E-64, 40 µM bestatin, 20 µM leupeptin and 0.8 µM aprotinin) for 20 min on ice. The solution was then centrifuged at 14,000 × *g* for 15 min; the supernatant was collected, and protein content was determined by the Bradford method. Samples (40 µg) were subjected to 10% SDS-PAGE, and proteins were transferred onto nitrocellulose membranes and blocked for one hour at room temperature with a solution of 5% non-fat milk in 0.1% Triton X-100. Overnight incubation at 4 °C was performed with anti-CHOP (1:100) and anti-tubulin (1:1000) and, then, with peroxidase-conjugated secondary antibody (1:3000) at room temperature for 1 hour. β-Tubulin was used as a loading control. Finally blots were subjected to a chemiluminescence detection kit (Super Signal West Pico; Pierce, Rockford, IL, USA).

## 4. Conclusions

In this work, we described the effect of a lipophilic extract obtained from *M. glacialis* against the human cancer cell lines, MCF-7 and SH-SY5Y. Evaluation of DNA synthesis revealed that both cell lines were markedly affected in a concentration-dependent way, the latter being more susceptible. We showed that the extract was responsible for two distinct effects: cell cycle arrest and apoptosis. We evaluated the contribution of the main compounds and demonstrated that while ergosta-7-dien-3-ol was responsible for the cell cycle arrest, palmitic acid was responsible for the apoptotic effect via the CHOP-mediated pathway of ER-stress. A proposed mechanism of the activity displayed by the extract can be found in Figure 9.
